# The Feasibility and Safety of Surgery in Patients Receiving Immune Checkpoint Inhibitors: A Retrospective Study

**DOI:** 10.3389/fonc.2017.00121

**Published:** 2017-06-12

**Authors:** Alexandra W. Elias, Pashtoon M. Kasi, John A. Stauffer, David D. Thiel, Dorin T. Colibaseanu, Kabir Mody, Richard W. Joseph, Sanjay P. Bagaria

**Affiliations:** ^1^Department of Surgery, Mayo Clinic, Jacksonville, FL, United States; ^2^Division of Hematology and Medical Oncology, Mayo Clinic, Jacksonville, FL, United States; ^3^Department of Urology, Mayo Clinic, Jacksonville, FL, United States

**Keywords:** immune checkpoint inhibitors, surgery, morbidity, serious adverse events, cancer

## Abstract

Immune checkpoint inhibitors (ICI) are revolutionizing care for cancer patients. The list of malignancies for which the Food and Drug Administration is granting approval is rapidly increasing. Furthermore, there is a concomitant increase in clinical trials incorporating ICI. However, the safety of ICI in patients undergoing surgery remains unclear. Herein, we assessed the safety of ICI in the perioperative setting at a single center. We conducted a retrospective review of patients who underwent planned surgery while receiving ICI in the perioperative setting from 2012 to 2016. We collected 30-day postoperative morbidity and mortality utilizing the Clavien–Dindo classification system. We identified 17 patients who received perioperative ICI in 22 operations. Patients were diagnosed with melanoma (*n* = 14), renal cell carcinoma (*n* = 2), and urothelial carcinoma (*n* = 1). Therapies included pembrolizumab (*n* = 10), ipilimumab (*n* = 5), atezolizumab (*n* = 5), and ipilimumab/nivolumab (*n* = 2). Procedures included cutaneous/subcutaneous resection (*n* = 6), lymph node resection (*n* = 5), small bowel resection (*n* = 5), abdominal wall resection (*n* = 3), other abdominal surgery (*n* = 3), orthopedic surgery (*n* = 1), hepatic resection (*n* = 1), and neurosurgery (*n* = 2). There were no Grade III–IV Clavien–Dindo complications. There was one death secondary to ventricular fibrillation in the setting of coronary artery disease. ICI appear safe in the perioperative setting, involving multiple different types of surgery, and likely do not need to be stopped in the perioperative setting. Further studies are warranted to confirm these findings.

## Introduction

Immune checkpoint inhibitors (ICI) are revolutionizing care for cancer patients. Programmed cell death ligand-1 (PD-L1), programmed death-1 (PD-1) receptor B7, and cytotoxic T-lymphocyte antigen-4 (CTLA-4) interactions are important immune escape mechanisms that allow tumor progression (1). ICI activate lymphocytes to eradicate tumor cells by recognizing these tumor-associated antigens. These ICI include CTLA-4 inhibitors, such as ipilimumab (Yervoy) and tremelimumab; PD-1 inhibitors, such as pembrolizumab (Keytruda) and nivolumab (Opdivo); and PD-L1 inhibitors, such as atezolizumab (Tecentriq) and durvalumab. They have demonstrated activity and efficacy in a variety of malignancies including, but not limited to, metastatic melanoma, lung cancer, renal cancer, bladder cancer, head and neck cancer, relapsed Hodgkin lymphoma, as well as a subset of gastrointestinal malignancies that are noted to be microsatellite instability high (2–6). The list of clinical indications for which the Food and Drug Administration is granting approval for the different ICI is rapidly increasing, and as a result, the number of clinical trials incorporating immunotherapy is rapidly increasing as well.

With the rising number of indications and patients on trials being treated with ICI, the perioperative safety and the risk of serious adverse events (SAEs) from surgery has not been adequately addressed. Other forms of systemic therapy, such as chemotherapy, have traditionally been held or stopped during the perioperative period due to their negative effects on wound healing, although multiple studies of late have shown some chemotherapy regimens to be safe in the perioperative period (7, 8). Typically, the duration of chemotherapy is approximately 2–4 weeks; and longer for biological agents such as bevacizumab that affect the vascular endothelial growth factor pathway. The mechanism of action and different spectrum of toxicity of ICI compared to chemotherapy and biological agents suggest that they may not significantly alter the perioperative course. Based on individual patient experiences, it is assumed that ICI may be safe in perioperative setting. Nonetheless, the safety of ICI in the perioperative setting for patients undergoing surgery remains unclear.

Ongoing studies that use immunotherapy in the neoadjuvant setting will address the safety and feasibility of ICI in the perioperative setting for early-stage cancer patients (9). However, there are no detailed published reports of perioperative morbidity, mortality, and readmissions in patients who continue ICI during the perioperative period. Herein, we present novel data assessing the feasibility and safety of ICI in the perioperative setting.

## Materials and Methods

We conducted a retrospective chart review of patients at Mayo Clinic Florida who underwent planned surgery while receiving ICI in the perioperative setting from 2012 to 2016. ICI drugs included pembrolizumab, ipilimumab, atezolizumab, and ipilimumab/nivolumab. We collected data from the electronic medical record on 30-day postoperative morbidity, mortality, and readmission rates. Complications were recorded and classified according to Clavien–Dindo grade (10). Grade I and II complications are minor complications that require minimal pharmacological intervention including blood transfusions and total parenteral nutrition. Grade III complications are those that require surgical, endoscopic, or radiological intervention. Grade IV complications are those that are life-threatening and require ICU admission, and Grade V complications are those that result in death. Indications for surgery were for either palliative (bleeding, pain, etc.) or non-palliative intent. Patients selected for a non-palliative intent were those who had persistent active or growing oligo-metastatic disease deemed resectable. All patients were treated a multidisciplinary decision setting. No statistical analyses or power calculations were required. Mayo Clinic Institutional Review Board approval was obtained prior to collecting identifiable patient information and performing analysis. The study was deemed as minimal risk and was exempt from obtaining informed consent from study patients.

## Results

We identified 17 patients who received perioperative ICI (Table 1). Ten patients were female and the mean age at time of surgery was 54 years. Cancers treated in this population included melanoma (*n* = 14), renal cell carcinoma (*n* = 2), and urothelial carcinoma (*n* = 1). Median hemoglobin was 12.8 g/dL (range 8.2–14.8 g/dL), median GFR was >60 mL/min/BSA, and median platelet count was 280/L (range 169–869/L). Therapies included pembrolizumab (*n* = 10), ipilimumab (*n* = 5), atezolizumab (*n* = 5), and ipilimumab/nivolumab (*n* = 2).

**Table 1:** Procedures, therapies, and complications.

Variable	*N* (%)
Types of procedures^a^	22
Cutaneous/subcutaneous	6 (27%)
Lymph node	5 (23%)
Bowel	5 (23%)
Abdominal wall	4 (18%)
Other abdominal	3 (14%)
Orthopedic	1 (5%)
Liver	1 (5%)
Brain	2 (9%)
Immune checkpoint inhibitors	
Pembrolizumab	10 (45%)
Ipilimumab	5 (23%)
Atezolizumab	5 (23%)
Ipilimumab/nivolumab	2 (9%)
Clavien–Dindo grade	
Grade I	4 (18%)
Grade II^b^	4 (18%)
Grade III	0 (0%)
Grade IV	0 (0%)
Grade V	1 (5%)

*^a^One procedure may fall into multiple categories*.

*^b^Total of five grade II complications during four procedures*.

Table 2 provides details on the 22 operations performed by 8 attending surgeons. Three patients had two procedures, and one patient had three procedures. Procedures included cutaneous/subcutaneous resection (*n* = 6), lymph node resection (*n* = 5), bowel resection (*n* = 5), abdominal wall resection (*n* = 3), other abdominal procedures (*n* = 3), neurosurgical procedures (*n* = 2), orthopedic procedures (*n* = 1), and hepatic resection (*n* = 1). Median estimated blood loss (EBL) was 23 mL (0–500 mL), median operative time was 129 min (22–404 min), and median length of stay was 1 day (0–11 days). Nine procedures (41%) were performed with palliative intent. Six procedures were performed in the outpatient setting.

**Table 2:** Patient characteristics, procedures, and immune checkpoint inhibitors (ICI) therapy.

Case no.	Disease	Procedure	ICI	Last dose of ICI before surgery, days	Last dose of ICI after surgery, days	Estimated blood loss, mL	Length of stay, days	Clavien–Dindo complications
1	Melanoma	Small bowel resection Colectomy	Pembrolizumab	28	N/A	400	11	Grade I—fat necrosis Grade II—DVT Grade II—anemia Grade V—death

2	Melanoma	Small bowel resection Soft tissue resection × 2 Pelvic lymph node dissection	Pembrolizumab	3	23	500	4	Grade II—anemia of chronic disease

3	Melanoma	Suboccipital craniotomy metastasectomy	Pembrolizumab	20	29	50	6	None

4	Melanoma	Laparoscopic resection of omental and mesenteric metastasis	Pembrolizumab	N/A	14	0	0	None

5	Melanoma	Pelvic lymph node dissection	Ipilimumab	32	N/A	50	3	None

6	Melanoma	Axillary lymph node dissection	Pembrolizumab	13	22	0	0	None

7	Melanoma	Laparoscopic non-anatomic liver resection	Ipilimumab	14	7	20	1	None

8	Melanoma	Frontal craniotomy metastasectomy	Ipilimumab	16	N/A	50	2	None
			Nivolumab					

9	Melanoma	Soft tissue resection × 2 Excision inguinal node	Ipilimumab	31	N/A	25	0	Grade I—seroma
			Nivolumab					

10	Melanoma	Laparoscopic small bowel resection	Pembrolizumab	12	9	50	3	None

11	Melanoma	Laparoscopic small bowel resection × 2	Ipilimumab	6	15	20	2	None

12	Melanoma	Soft tissue resection	Ipilimumab	20	1	3	0	Grade II—DVT

13	Melanoma	Axillary lymph node dissection	Ipilimumab	9	34	50	1	None

14	Melanoma	Soft tissue resection	Pembrolizumab	7	14	2	0	None

15	Melanoma	Adrenalectomy	Pembrolizumab	1	21	75	1	None

16	Melanoma	Laparoscopic small bowel resection	Pembrolizumab	N/A	30	5	2	Grade I—superficial wound separation

17	Melanoma	Soft tissue resection	Pembrolizumab	7	14	2	0	None

18	Urothelial	Nephroureterectomy	Atezolizumab	16	26	500	4	Grade II—anemia requiring transfusion

19	Renal cell	Humerus curettage	Atezolizumab	21	29	150	1	None

20	Renal cell	Laparoscopic abdominal wall resection	Atezolizumab	8	13	15	1	None

21	Renal cell	Laparoscopic abdominal wall resection	Atezolizumab	36	27	0	0	Grade I—superficial wound separation

22	Renal cell	Laparoscopic abdominal wall resection	Atezolizumab	30	33	5	1	None

The median duration of time between last preoperative ICI dose and surgery was 16 days (1–32 days), and the median duration of time between surgery and first postoperative ICI dose was 18 days (1–34 days). The number of procedures that were performed 1–7 days of last ICI dose was 8, 8–14 days of last ICI dose was 5, and 15–21 days of last ICI dose was 3.

There were no Grade III–IV Clavien–Dindo complications or readmissions within 30 days of surgery. Nine Grade I and II complications were noted in nine procedures, most commonly superficial wound complications or anemia requiring blood transfusion. One patient who underwent small bowel and colon resection for melanoma metastases while undergoing treatment with pembrolizumab had a history of stent placement for coronary artery disease status died of ventricular fibrillation on postoperative day 5.

There were five bowel resection procedures that resulted in seven bowel anastomoses. There were no anastomotic leaks and one procedure resulted in a Grade I superficial wound separation. There were five major non-bowel surgical procedures. The neurosurgical procedures included resection of a cerebellar metastasis through a suboccipital craniotomy (pembrolizumab administered 20 days before and 29 days after) and resection of a frontal metastasis through a frontal craniotomy (ipilimumab/nivolumab administered 16 days before). The orthopedic procedure involved curettage of metastasis to the humerus (atezolizumab administered 21 days before and 29 days after). The hepatic procedure was a laparoscopic non-anatomic resection of the segment 6 (ipilimumab administered 14 days before and 7 days after). All five major non-bowel procedures had no complications.

## Discussion

Immunotherapy is rapidly becoming integrated into the multidisciplinary care of a wide range of malignancies. To treat these complex conditions, it is critical to understand whether ICI should be held for or delay surgery. Furthermore, as ICI are now being incorporated in various clinical trials in the neoadjuvant setting (NCT02930902, NCT02735239, NCT03003637), natural concerns have been raised concerning the safety of surgery while patients receive ICI therapy. Therefore, for the medical as well as a surgical oncology community, it is important to understand if such patients are at higher risk of morbidity and mortality, and whether or not ICI need to be held for or should delay surgery.

Our patient population consisted of a small group of adult patients with metastatic disease, for whom a multidisciplinary decision was made at our tertiary care center to resect sites of metastasis that caused symptoms or remained active following a mixed response to ICI therapy. Patients underwent planned operations that ranged in complexity (simple soft tissue resections vs. liver resections) and surgical discipline (surgical oncology, neurosurgery, and orthopedic oncology). We chose to classify complications by Clavien–Dindo grade, as it is an accepted standard system used by surgeons to measure postoperative complications. In this system, complications are graded by the therapeutic interventions required, in order to provide a more objective and consistent assessment of severity. In general, grade I and II complications are considered to be surmountable, while grade III and IV complications are considered to be more substantial, due to their association with higher mortality, patient stress, and increased resource consumption (10).

The majority of procedures (*n* = 13) were done within 14 days of receipt of ICI therapy. EBL, duration of surgery, and length of stay were all as anticipated based on the scope of the procedures. We collected data on morbidity, mortality, and readmissions from the charts of these patients and identified no Grade III and IV complications or readmissions. One death (Grade V) was noted and does not appear to be secondary to ICI, given the patient’s cardiac history.

Immune checkpoint inhibitors modulate interactions between T cells and either antigen-presenting cells or tumor cells in order to stimulate antitumor activity (1). CTLA-4 is a negative regulator of T-cell activation, thus antibodies to CTLA-4, such as ipilimumab, are intended to activate the immune system against tumors by preventing T-cell repression. This effect is not limited to the tumor microenvironment. On the other hand, PD-L1, which is expressed in high levels by some tumors, suppresses the immune response through interaction with PD-1 within the tumor microenvironment. Anti-PD-1 and anti-PD-L1 antibodies block this interaction, thus reactivating the immune response to the tumor. These drugs may have less widespread T-cell activation, due to limited expression of PD-L1 in normal tissue. While the toxicity profile is favorable compared to traditional chemotherapy, the immune boosting mechanism of these monoclonal antibodies can produce severe autoimmune adverse effects, such as pneumonitis, arthralgia, pyrexia, colitis, dermatitis, hepatitis, endocrinopathy, and neuropathy (2, 3). The complications experienced during our study do not appear to be related to the toxicity profile of these drugs.

Review of the literature identifies small studies and abstracts presented at conferences that attempt to determine the safety of ICI. Baker and colleagues reviewed publications that reported on patients receiving ipilimumab undergoing surgery. They described a total of seven patients who underwent a surgical intervention and reported no SAEs attributable to ICI in various studies. Additionally, they noted no complications in three patients who were operated at their institution while receiving ICI. Their conclusion was that there is no reason to withhold or delay surgery for patients receiving ipilimumab therapy. In a study presented at the American Society of Clinical Oncology in 2012, Gyorki and colleagues reported on 23 patients who underwent 34 operations within 30 days of receiving a dose of ipilimumab and identified no SAEs. They reported ICI to be safe without affecting wound healing even in patients undergoing bowel surgery (11).

As noted, there are now ongoing studies incorporating the use of ICI in the neoadjuvant setting and specifically address the safety and feasibility of ICI in the perioperative setting for early-stage cancer patients. A pilot study presented at the European Society for Medical Oncology in 2016 reported on 16 patients with early-stage lung cancer who received two doses of nivolumab at 4 and 2 weeks prior to surgical resection of the tumor (9). There were no SAEs attributable to the ICI and no delays to getting the surgery.

This study included a wide variety of surgical procedures and involved multiple surgical disciplines and therefore may broadly apply to multiple scenarios. While our study is limited by its small size and retrospective nature, our data and review of literature provide valuable evidence that continuing ICI without interruption during the perioperative period may be feasible and safe. Our results suggest that for even major operations, ICI likely does not need to be stopped in anticipation of surgery or for that matter should not delay surgery and that clinical trials that utilize neoadjuvant or adjuvant ICI may not need to delay the time between surgery and ICI. Further studies will be needed to confirm our findings.

## Author Contributions

AE contributed to collection, analysis, and interpretation of the data; drafting and critical revision of the article; and generation of the figures. PK contributed to the analysis and interpretation of the data and drafting of the article. JS, DT, DC, and KM contributed to the experiments, analysis and interpretation of the data, and critical revision of the article. RJ contributed to the conception and design of the study, experiments, analysis and interpretation of the data, and critical revision of the article. SB contributed to the conception and design of the study, experiments, analysis and interpretation of the data, and drafting and critical revision of the article. All authors gave final approval of the article.

## Conflict of Interest Statement

The research was conducted in the absence of any commercial or financial relationships that could be construed as a potential conflict of interest.
